# Effect of anakinra on arthropathy in CINCA/NOMID syndrome

**DOI:** 10.1186/1546-0096-8-9

**Published:** 2010-03-16

**Authors:** Takako Miyamae, Yutaka Inaba, Gen Nishimura, Masako Kikuchi, Takayuki Kishi, Ryoki Hara, Utako Kaneko, Toshihiko Shinoki, Tomoyuki Imagawa, Shumpei Yokokta

**Affiliations:** 1 Department of Pediatrics, Yokohama City University, Yokohama, Japan; 2 Department of Orthopedics, Yokohama City University, Yokohama, Japan; 3 Department of Radiology, Tokyo Metropolitan Kiyose Children's Hospital, Tokyo, Japan

## Abstract

CINCA/NOMID is an autoinflammatory disorder characterized by the triad of neonatal onset of cutaneous symptoms, chronic meningitis, and recurrent fever and it presents with distinctive osteoarthropathy, synovitis mainly of the large joints and overgrowth of epimetaphyseal cartilage, particularly of the long bones. The cartilage overgrowth eventually causes osseous overgrowth and deformity that persists beyond skeletal maturity and leads to limb length discrepancy, joint contracture, and early degenerative arthropathy. Autoinflammation in CAPS/NOMID has been proven to derive from excessive release of interleukin-1 (IL-1). It has been well documented that the IL-1 receptor antagonist anakinra (Kineret(R)) helps mitigate systemic inflammation in the disorder. However, a general consensus has not been reached on its beneficial effect on osteoarthropathy. The case of a girl with CINCA/NOMID syndrome who showed dramatic improvement of osteoarthropathy after anakinra treatment is reported. A 4-year-old girl suffered at the age of 10 months from a generalized urticarial skin lesion with recurrent episodes of fever and growth disorder. Blood examination revealed persistent massive neutrophilia, anemia and intense acute phase response. She manifested knee joint swelling with limited ROM when she was 20 months old and was diagnosed as being CINCA/NOMID based on characteristic findings of radiograph despite negative CIAS1 mutation. Radiological examination demonstrated metaphyseal fraying and cupping and widening of the growth plate in the distal femur. MR imaging showed mottled gadolinium enhancement at the chondrosseous junction. Neither significant joint effusion nor synovitis was identified. At 2 years and 7 months of age, anakinra, 2 mg/kg/day given by regular daily subcutaneous injections, was started. A few days after the initiation of the treatment, her clinical symptoms and laboratory findings of active inflammation were promptly alleviated. She was not able to walk unaided prior to the treatment, but she walked independently 1 month after the treatment. Follow-up radiographs and MR imaging showed that growth plate widening and gadolinium enhancement at the chondrosseous junction were less conspicuous. Furthermore, longitudinal growth of the femur and tibia was identified during 20 months of observation.

## Background

CINCA/NOMID is an autoinflammatory disorder characterized by the triad of neonatal onset of cutaneous symptoms, chronic meningitis, and recurrent fever [1-4] Since many cases are attributed to heterozygous gain-of-function mutations in *NLRP3 (CAIS1)*, the gene encoding cryopyrin [5,6], it is classified into cryopyrin-associated periodic syndromes (CAPS). CAPS include three allelic variants, ranging in order of increasing severity from Familial Cold Auto-inflammatory Syndrome (FCAS), previously termed Familial Cold Urticaria, through Muckle-Wells Syndrome (MWS) to Chronic Infantile Neurologic Cutaneous Articular Syndrome or Neonatal-Onset Multisystem Inflammatory Disease (CINCA/NOMID) [7,8]. However, CINCA/NOMID may be heterogeneous, and only 60% of affected individuals have *NLRP3 *mutations.

CINCA/NOMID presents with distinctive osteoarthropathy, mainly of the large joints and overgrowth of epimetaphyseal cartilage, particularly of the long bones. Histological examination for overgrown cartilage shows complete disorganization of the cartilage cell columns and irregular metachromasia of the cartilage substance, but no inflammatory cell infiltrates [4]. The cartilage overgrowth eventually causes osseous overgrowth and deformity that persists beyond skeletal maturity and leads to limb length discrepancy, joint contracture, and early degenerative arthropathy [3,9,10] In particular, osteocartilaginous overgrowth in the patella and distal femur is so characteristic that its presence warrants a diagnosis of CINCA/NOMID.

Autoinflammation in CAPS has been proven to derive from excessive release of interleukin-1β (IL-1β) [11]. Interestingly, deficiency of the IL-1 receptor antagonist due to mutations of *IL1RN *gives rise to the phenotype sharing some features with CINCA/NOMID but with some clinical peculiarities regarding skin and bone manifestations [12]. It has been well documented that the IL-1 receptor antagonist anakinra (Kineret^®^) helps mitigate systemic inflammation in both disorders [12-14]. However, a general consensus has not been reached on its beneficial effect on osteoarthropathy. The case of a girl with CINCA/NOMID syndrome who showed dramatic improvement of overgrowth osteoarthropathy after anakinra treatment is reported.

## Case presentation

**Figure 1:**
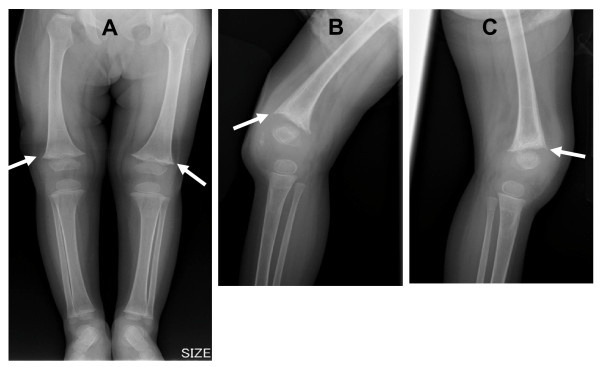
**Radiographs at 24 months of age.**  (A: frontal view of the lower extremities, B: lateral view of the right knee, C: lateral view of the left knee) show metaphyseal fraying and cupping and widening of the growth plate in the distal femur (arrow).

**Figure 2:**
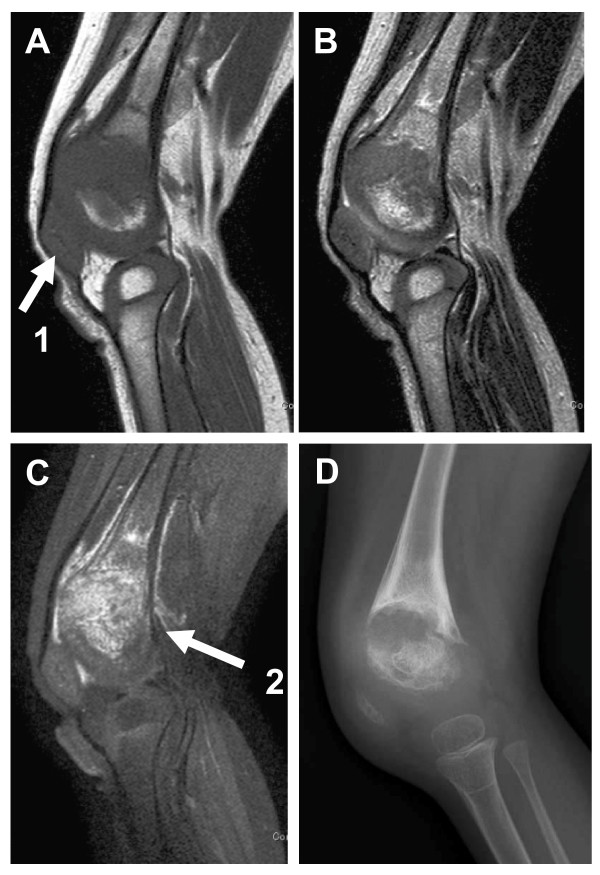
**Sagittal MR images and lateral radiograph of the left knee at 2 years and 7 months of age, prior to introduction of anakinra.**  (A: MRI T1-weighted, B: MRI T2-weighted, C MRI T1-weighted with fat suppression and gadolinium-enhancement, D radiograph): T1- and T2-weighted MR images show widening of the growth plate (arrow 1), and a gadolinium-enhanced MR image shows mottled enhancement at the chondroosseous junction (arrow 2).

**Figure 3:**
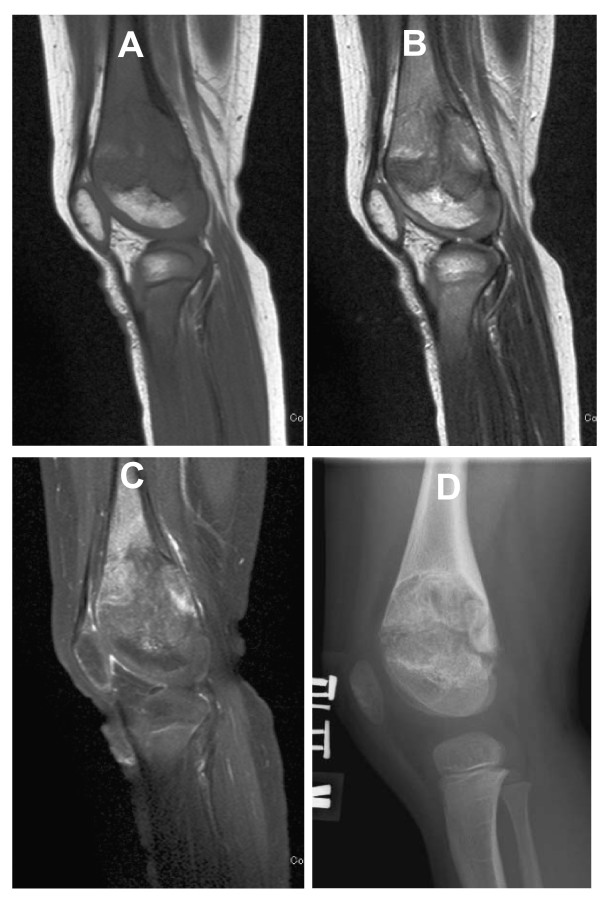
**MR images and radiograph of the left knee at the age of 4 years and 3 months, after more than 20 months of anakinra treatment**. (MRI A: T1-weighted, B: MRI T2-weighted, C: MRI T1-weighted with fat suppression and Ga enhancement, D radiograph). The growth plate widening previously seen (Figure 2) has alleviated with anakinra treatment. A gadolinium-enhanced MR image demonstrates less conspicuous enhancement at the chondroosseous junction after the anakinra treatment.

The girl was born by normal vaginal delivery following an unremarkable pregnancy. The parents were healthy and nonconsanguineous. At 10 months of age, the girl presented with recurrent episodes of fever and growth disorder associated with a generalized, maculopapular, urticaria-like skin rash. Blood examination revealed massive neutrophilia, anemia, and intensely elevated acute phase reactants. Antibiotic treatment failed to alleviate the clinical symptoms. At 20 months of age, she developed knee joint swelling with limited ROM resulting in problems with standing and walking. Radiological examination demonstrated metaphyseal fraying and cupping and widening of the growth plate in the distal femur (Figure 1). MR imaging showed mottled gadolinium enhancement at the chondrosseous junction (Figure 2). Neither significant joint effusion nor synovitis was identified. She was diagnosed as having CINCA/NOMID on clinical and radiological grounds; however, analysis of cerebrospinal fluid (CSF) showed neither pleocytosis nor increased protein levels, and a molecular examination did not show *NARLP3 *mutations. At 2 years and 7 months of age, anakinra, 2 mg/kg/day given by regular daily subcutaneous injections, was started. A few days after the initiation of the treatment, her constitutional symptoms, fever and urticaria-like rash, and acute-phase reactant levels were promptly alleviated. She was not able to walk unaided prior to the treatment, but she walked independently 1 month after the treatment. Follow-up radiographs and MR imaging (Figure 3) showed that growth plate widening and gadolinium enhancement at the chondrosseous junction were less conspicuous. Furthermore, longitudinal growth of the femur and tibia was identified during 20 months of observation (Data not shown).

## Discussion

Although no *NARLP3 *mutations were found in the present case, a diagnosis of CINCA/NOMID was warranted on clinical and radiological grounds. Her dermatological manifestation differed from that of deficiency of the IL-1 receptor antagonist (urticaria-like skin rash vs. pyoderma). Her clinical and radiological evolution following anakinra treatment implied that the therapy was effective not only in mitigating clinical symptoms of autoinflammation but also in preventing progression of osteoarthropathy. The beneficial effect of anakinra on arthropathy has been controversial. The case represented here indicated that the earlier anakinra was initiated, the better arthropathy was overcome. If the treatment had not been introduced, the osteoarthropathy eventually would have led to severe joint deformations, limb length discrepancy, and growth retardation, as previously reported. Once bone deformations develop in patients with CINCA/NOMID, they are very difficult to reverse [14]. However, the tragic consequences can be prevented by early medical intervention in this case.

The imaging findings in the present girl included widening of the growth plate and gadolinium enhancement at the chondrosseous junction. These findings are in a broad sense termed ''metaphyseal dysplasia'', and are consistent with the known histological findings in CINCA/NOMID. According to a previous report, the histological findings for biopsy specimens from a ''bony mass'' were 1) disorganized cartilage at the growth plate and 2) thin metaphyseal trabeculae mixed with relatively acellular cartilage, fibrous tissues, and foci of calcification [10] The latter finding may correspond with gadolinium enhancement of the chondrosseous junction. In the present case, the anakinra treatment provided remodeling of the ''metaphyseal dysplasia'' with alleviation of gadolinium enhancement. This fact suggests that osteoarthropathy in CINCA/NOMID is caused by overproduction of IL-1β, as are the symptoms of autoinflammation and disappearance of them by specific receptor antagonist of IL-1β.

Osteoarthropathy is significant in approximately 60% of CINCA/NOMID patients, and it is more prevalent in cases with deficiency of the IL-1 receptor antagonist. The mechanism to explain why the excessive production of IL-1β causes distinctive osteocartilaginous overgrowth in CINCA/NOMID still remains unclear. Though it has been reported that IL-1β exerts inhibitory effects on murine ATDC5 chondrocyte dynamics and metatarsal longitudinal growth [15], recent observation suggested that overgrowth arthropathy in CINCA/NOMID is not driven by IL-1β, but such overgrowth may be due to abnormal apoptosis at the site of enchondral ossification as NALP3 is expressed in cartilage [16,17].

## Conclusion

This report indicated the importance of earlier indication of anakinra for better outcome of arthropathy in CINCA/NOMID syndrome. Once once bone deformations develop in the patients, they are very difficult to reverse even with anakinra.

## Consent

Written informed consent was obtained from the patient for publication of this case report and any accompanying images. A copy of the written consent is available for review by the Editor in Chief of this journal.

## Competing interests

The authors declare that they have no competing interests.

## Authors' contributions

TM drafted the manuscript and participated in its design. MK, TK, RH, UK, TS and TI participated in drafting of the manuscript and participated in its design. YI and GN participated in the drafting of the manuscript and supplied the radiological images used for the manuscript. SY conceived of the case report, participated in drafting the manuscript and gave final approval for the version to be submitted for publication.
